# Supplementary material for: The Conservation of Low Complexity Regions in Bacterial Proteins Depends on the Pathogenicity of the Strain and Subcellular Location of the Protein
Source: Genes (Basel). 2021 Mar 22;12(3):451. doi: 10.3390/genes12030451 (PMC8004648; doi:10.3390/genes12030451)

(a)

```
R9LP70     LSDKAAAAFPLTVERKQQYLETTGINDRLIMLLQDFEKEKEMSEVEKKINDTVKERIDQG
A0A418MLU7 LADAIAAQLPLKIKEKQEILESFNVSERLDRVLSSLADEREVLELEKEIHQRVRKQMEKT
LON_ECOLI  LADTIAAHMPLKLADKQSVLEMSDVNERLEYLMAMMESEIDLLQVEKRIRNRVKKQMEKS
           *:*  ** :**.:  **. **  .:.:**  ::  : .* :: ::** *.: *.:.:::

R9LP70     QKDYYLREKIHAIREELGDIVETDKDAESIRKRLAEEPYPEYIKDKVKEELMRYEMLPQA
A0A418MLU7 QKEYYLREQMKAIQRELGDKEGRQGEIDELRDKMANLQLPELVAMRVDKEINRLERIPIS
LON_ECOLI  QREYYLNEQMKAIQKELGEMDDAPDENEALKRKIDAAKMPKEAKEKAEAELQKLKMMSPM
           *.:***.*:::**. ***:      : : :. .:     *:    ... *: . : :.

R9LP70     NGESGVIKTYIDWLMDLPWWQTTKDNEDLESAQNILDEDHYGLAKVKERIMEYLAVKQMT
A0A418MLU7 SAEGTVARTFIDWLLDLPWTAKSVSIIDLGRAQRVLDAHHYGLEKVKDRIIDYLAVQKLT
LON_ECOLI  SAEATVVRGYIDWMVQVPWNARSKVKKDLRQAQEILDTDHYGLERVKDRILEYLAVQSRV
           ..*. * . :***::::**   :    **  ** :**  **** .**:**::****:. .
```

(b)

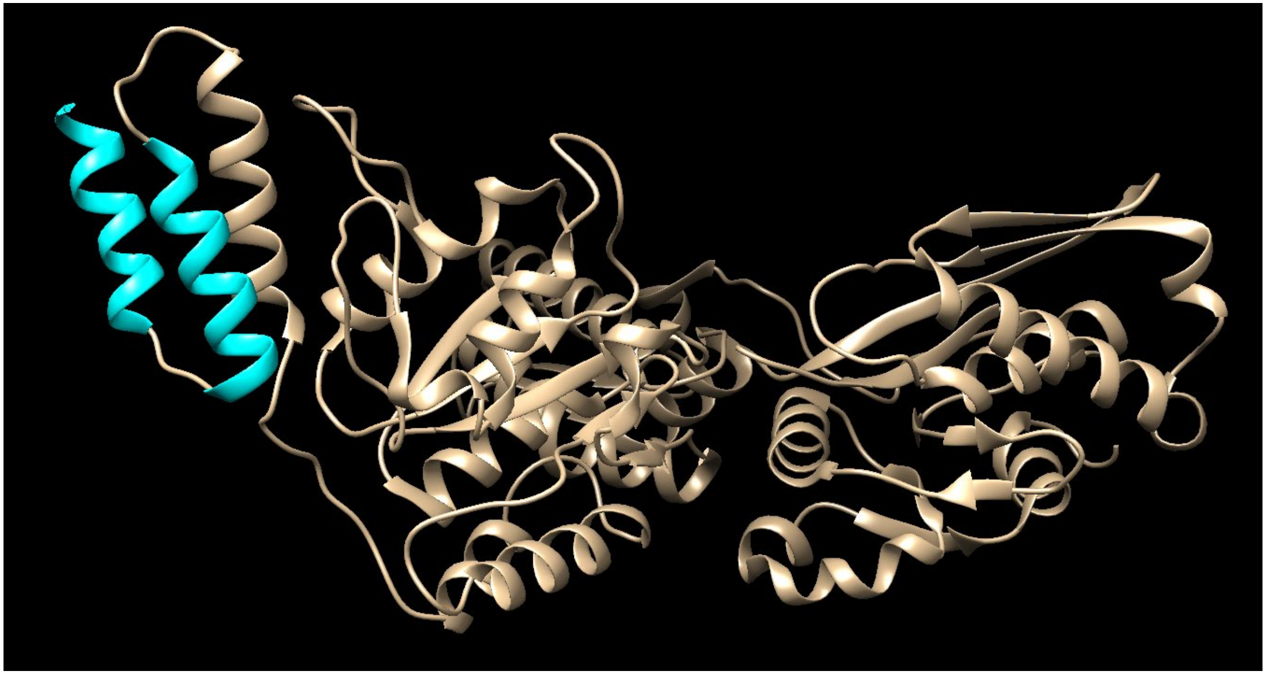

Supplement: Supplementary file 1 [file genes-12-00451-s001.zip › LCRstrains_SupplMaterial_v2/SupplFigS5.pdf]
