# Supplementary figures and images for: The Conservation of Low Complexity Regions in Bacterial Proteins Depends on the Pathogenicity of the Strain and Subcellular Location of the Protein
Source: Genes (Basel). 2021 Mar 22;12(3):451. doi: 10.3390/genes12030451 (PMC8004648; doi:10.3390/genes12030451)

**(a)**

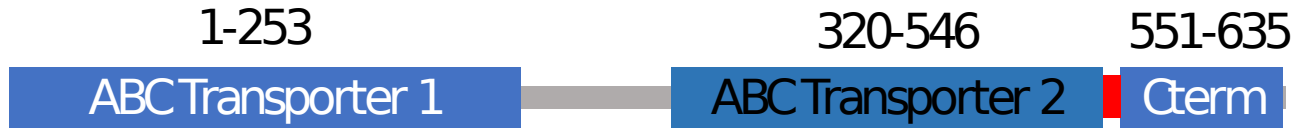

**(b)**

547-550

|                   |                        |
|-------------------|------------------------|
| <b>UUP_ECOLI</b>  | <b>VKKTEEAAA AKAET</b> |
| <b>A0A0H3JCY6</b> | <b>VKKNEEPAAPKAET</b>  |

Supplement: Supplementary file 1 [file genes-12-00451-s001.zip › LCRstrains_SupplMaterial_v2/SupplFigS4.pdf]

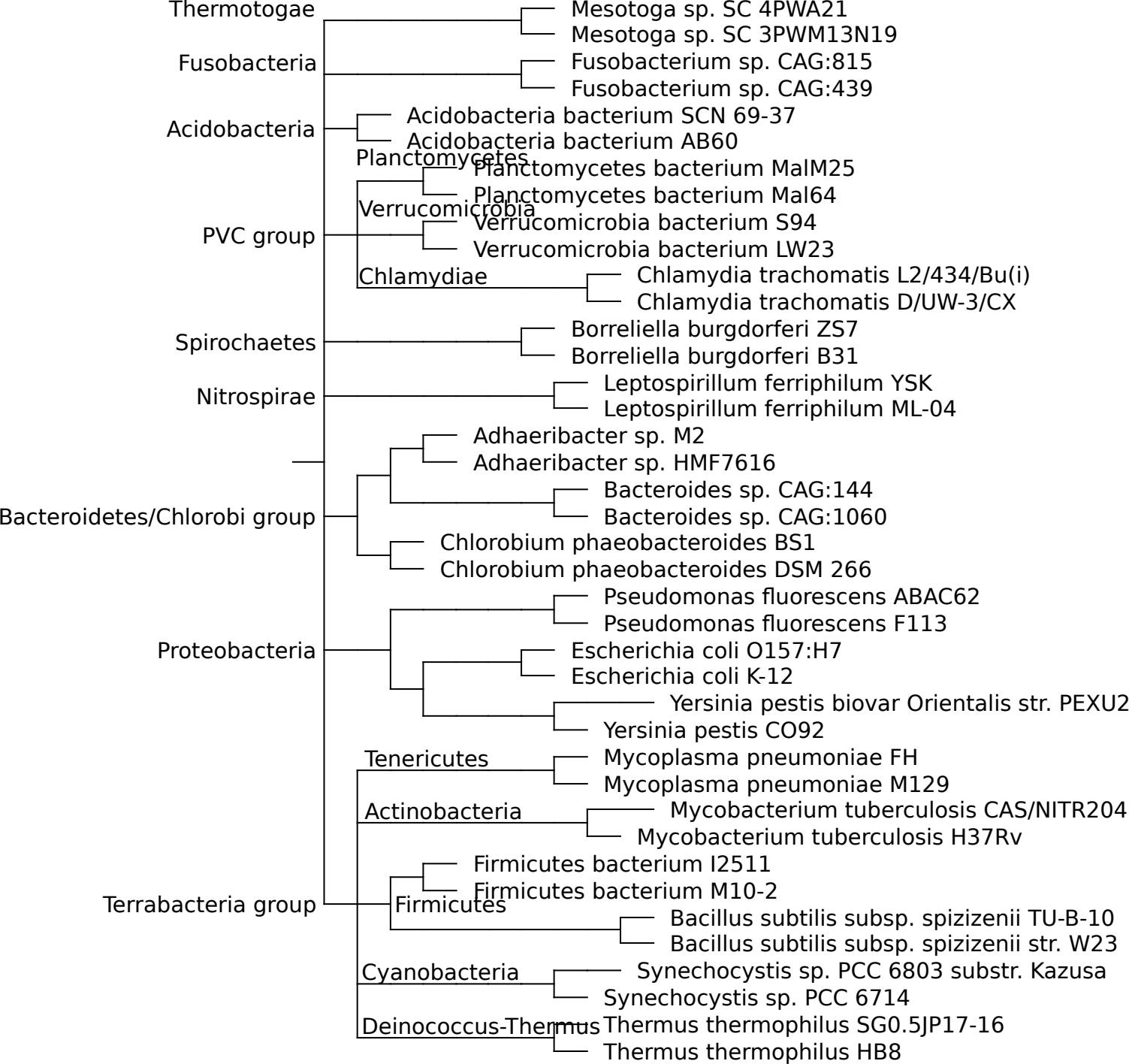

Supplement: Supplementary file 1 [file genes-12-00451-s001.zip › LCRstrains_SupplMaterial_v2/SupplFigS1.pdf]

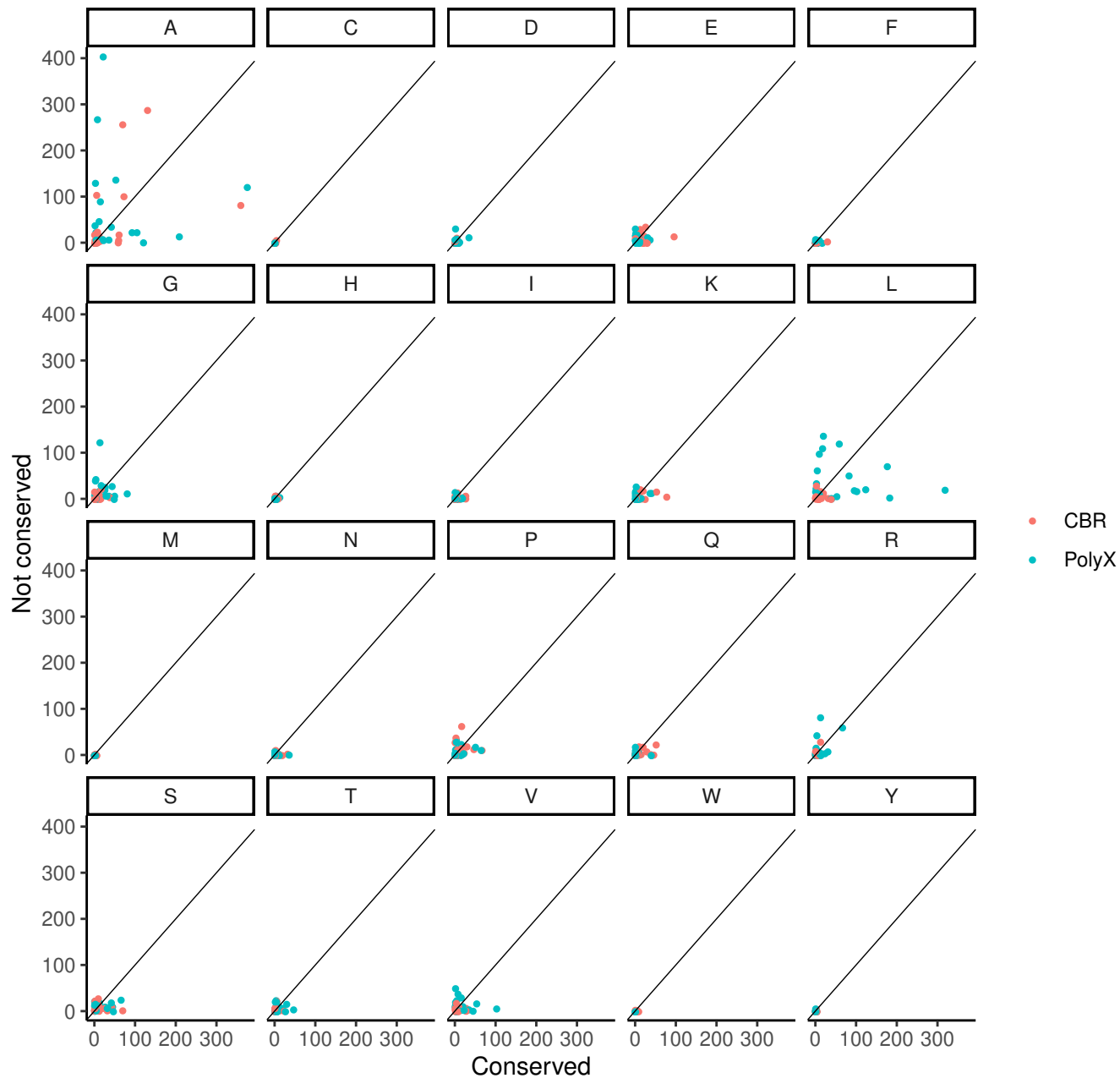

Supplement: Supplementary file 1 [file genes-12-00451-s001.zip › LCRstrains_SupplMaterial_v2/SupplFigS3.pdf]

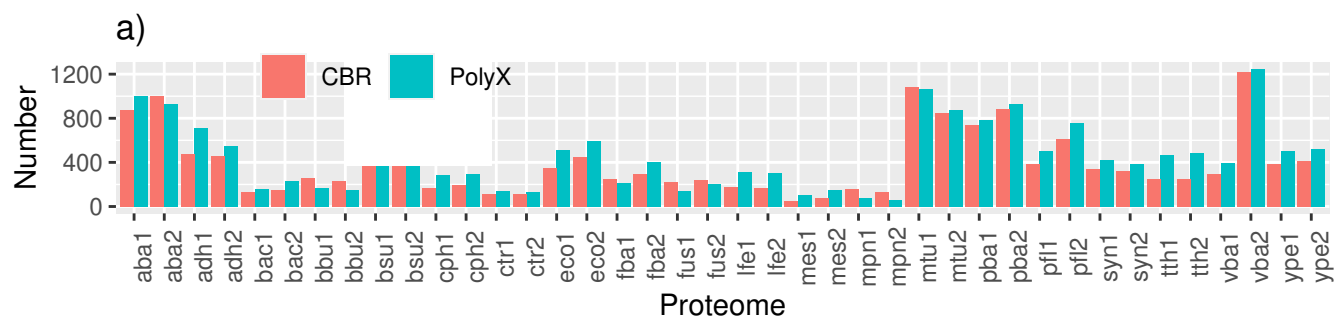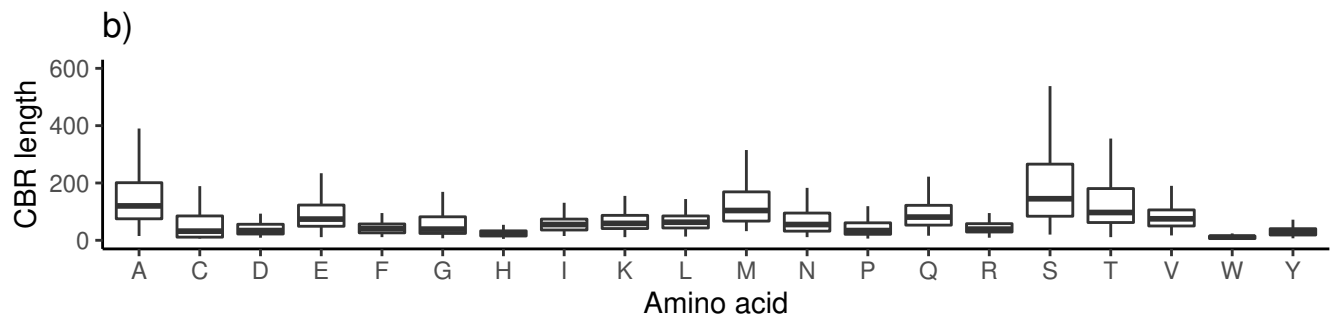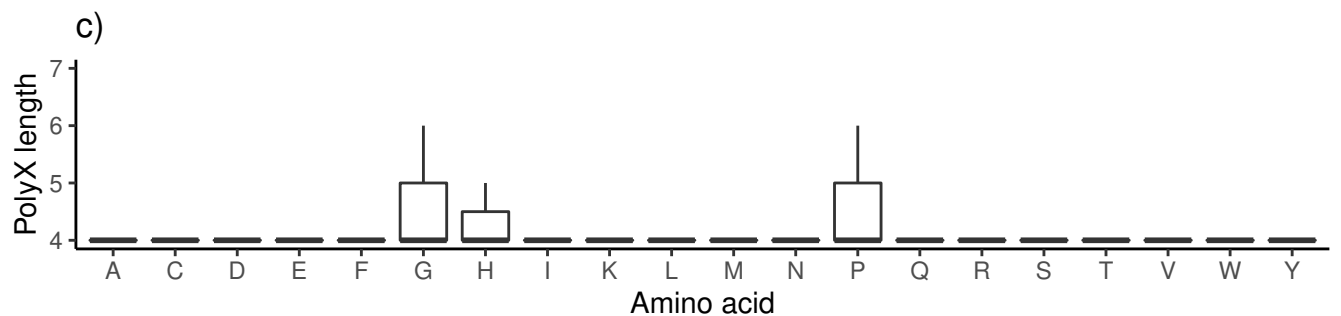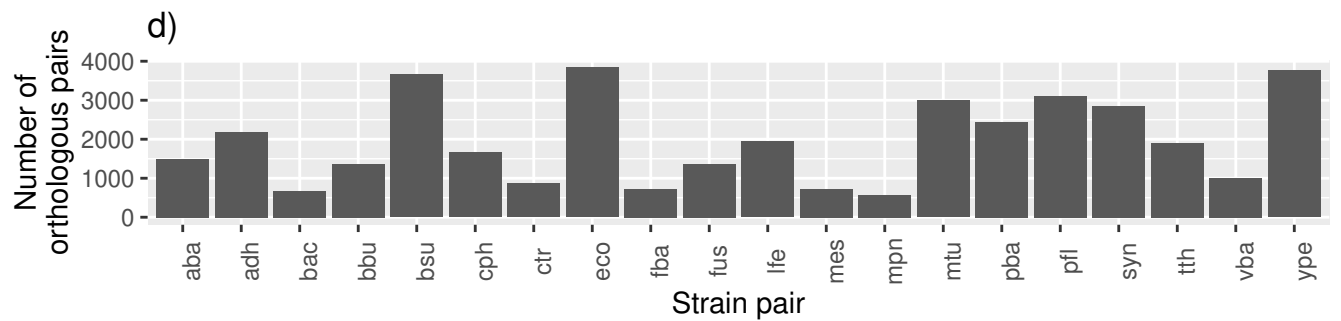

Supplement: Supplementary file 1 [file genes-12-00451-s001.zip › LCRstrains_SupplMaterial_v2/SupplFigS2_v2b.pdf]
